# Sequence determinants in the cathelicidin LL-37 that promote inflammation *via* presentation of RNA to scavenger receptors

**DOI:** 10.1016/j.jbc.2021.100828

**Published:** 2021-05-26

**Authors:** Nikhil N. Kulkarni, Alan M. O’Neill, Tatsuya Dokoshi, Elizabeth W.C. Luo, Gerard C.L. Wong, Richard L. Gallo

**Affiliations:** 1 Department of Dermatology, University of California, San Diego, San Diego, California, USA; 2 Department of Bioengineering and Chemistry, University of California Los Angeles, Los Angeles, Louisiana, USA

**Keywords:** antimicrobial peptides, cathelicidin, interferon, cytokines, skin, GAS, group A *Streptococcus*, GO, Gene Ontology, HDMEC, human dermal microvascular endothelial cell, I20P, proline at isoleucine position 20, IL-6, interleukin 6, IRF7, interferon regulatory factor 7, LDH, lactate dehydrogenase, LL-34, 34-amino acid peptide, LL-37, 37-amino acid peptide, MAVS, mitochondrial antiviral signaling, MIC, minimum inhibitory concentration, NHEK, normal human epidermal keratinocyte, qRT-PCR, quantitative RT-PCR, RIGI, retinoic acid–inducible gene I, SAXS, small-angle X-ray scattering, SR, scavenger receptor, TBK1, TANK-binding kinase 1, UCSD, University of California, San Diego

## Abstract

Cathelicidins such as the human 37-amino acid peptide (LL-37) are peptides that not only potently kill microbes but also trigger inflammation by enabling immune recognition of endogenous nucleic acids. Here, a detailed structure–function analysis of LL-37 was performed to understand the details of this process. Alanine scanning of 34-amino acid peptide (LL-34) showed that some variants displayed increased antimicrobial activity against *Staphylococcus aureus* and group A *Streptococcus*. In contrast, different substitutions clustered on the hydrophobic face of the LL-34 alpha helix inhibited the ability of those variants to promote type 1 interferon expression in response to U1 RNA or to present U1 to the scavenger receptor (SR) B1 on the keratinocyte cell surface. Small-angle X-ray scattering experiments of the LL-34 variants LL-34, F5A, I24A, and L31A demonstrated that these peptides form cognate supramolecular structures with U1 characterized by inter-dsRNA spacings of approximately 3.5 nm, a range that has been previously shown to activate toll-like receptor 3 by the parent peptide LL-37. Therefore, while alanine substitutions on the hydrophobic face of LL-34 led to loss of binding to SRs and the complete loss of autoinflammatory responses in epithelial and endothelial cells, they did not inhibit the ability to organize with U1 RNA in solution to associate with toll-like receptor 3. These observations advance our understanding of how cathelicidin mediates the process of innate immune self-recognition to enable inert nucleic acids to trigger inflammation. We introduce the term “innate immune vetting” to describe the capacity of peptides such as LL-37 to enable certain nucleic acids to become an inflammatory stimulus through SR binding prior to cell internalization.

Cathelicidins are a family of antimicrobial and immunomodulatory peptides found across diverse species and produced by many cell types (1). In humans, only a single cathelicidin gene called *CAMP* is known, and it encodes a mature 37-amino acid peptide (LL-37) that is cationic, alpha helical, and amphipathic. LL-37 has multiple biological actions in addition to its capacity to kill bacteria. These include chemotaxis, wound healing, lipopolysaccharide neutralization, and angiogenesis (2, 3). Cathelicidin is an important component in neutrophil extracellular traps that promote innate and adaptive immune responses (4). The active LL-37 peptide is processed from proprotein hCAP18 by protease-mediated cleavage with neutrophil proteinase 3 and epithelial kallikreins like kallikrein gene 5 (5, 6). Previous studies have shown that human autoinflammatory diseases like rosacea or psoriasis are promoted by the presence of excess LL-37 within the skin (5).

Inflammation present in human diseases such as psoriasis and rosacea is amplified in part through the capacity of LL-37 to enhance the recognition of self nucleic acids. One example is noncoding U1 dsRNA that is released from damaged cells, leading to activation of endosomal or cytosolic pattern recognition receptors with subsequent release of inflammatory cytokines like tumor necrosis factor alpha or type 1 interferons (7, 8, 9). Enhanced LL-37 expression has also been implicated in the pathophysiology of several other diseases like breast cancer, atherosclerosis, Alzheimer's disease, and lupus (5, 10, 11, 12), thus suggesting that the autoinflammatory activity of LL-37 may be important to a wide range of human diseases. Cationic amino acid residues and the alpha helix are essential for antimicrobial activity of LL-37 (13), and rational design of peptides derived from LL-37 has been used to enhance antimicrobial action against both gram-positive and gram-negative bacteria, viruses, and trophozoites with reduced cytotoxicity or expand its inflammatory action without antimicrobial function (14, 15, 16, 17, 18, 19, 20, 21, 22). However, much less is known about how LL-37 promotes disease through its inflammatory action.

LL-37 has been shown to exhibit its antibacterial and antiviral activities by membrane destabilization, but its immunomodulatory activities are less well understood and appear dependent on its capacity to trigger activation of pattern recognition and cell surface receptors like toll-like receptor 2, epidermal growth factor receptor, and formyl peptide receptor 2 (23, 24, 25, 26). LL-37 has also been shown to potentially act as an autoantigen by triggering specific CD4- and CD8-specific humoral responses (27, 28, 29). However, although evidence for receptor activation by LL-37 has been demonstrated, these interactions have not clearly established specific binding to receptor kinases or a clear mechanism of action for this important peptide.

In the present study, we employed a systematic alanine scan of a bioequivalent version of LL-37 to investigate which amino acid residues are necessary to permit it to enable immune recognition self nucleic acids such as U1 dsRNA. We refer to this process as “innate immune vetting” as the presence of LL-37 in a tissue or cell culture enables otherwise noninflammatory nucleic acid fragments to become innately proinflammatory. Our findings uncover a previously unknown structural element necessary for this function and increase understanding of how LL-37 regulates tissue inflammation.

## Results

### Sequential substitution of LL-34 with alanine reveals the critical amino acids necessary for the innate vetting activity

**Figure 1:**
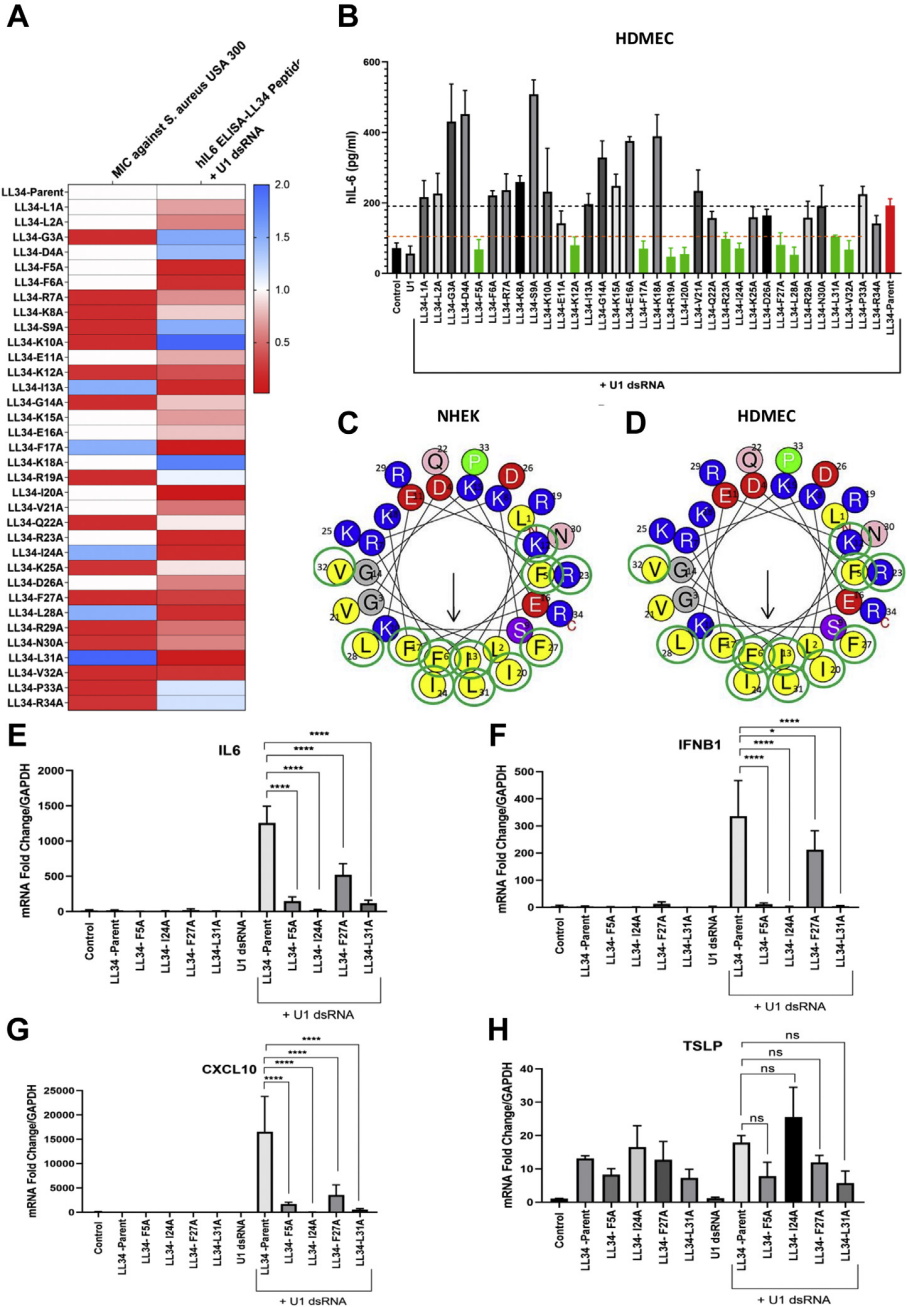
**Sequential alanine substitution in LL-34 reveals amino acid residues critical for innate vetting activity *in vitro*.***A*, normal human keratinocytes (NHEKs) were treated with either U1 dsRNA (2.5 μg/ml) or a combination of U1 dsRNA (2.5 μg/ml) and LL-34 peptides (LL-34 L1A and R34A and LL-34 parent) at 2 μM for 18 h. Protein levels of human IL-6 were analyzed from cell supernatant with ELISA (n = 3). Corresponding MIC values of LL-34 peptides L1A–R34A against *Staphylococcus aureus* USA300 are shown in the heat map. *B*, human dermal endothelial cells (HDMECs) were treated with either U1 dsRNA (2.5 μg/ml) or a combination of U1 dsRNA (2.5 μg/ml) and LL-34 peptides (Pep 1–Pep 34 and Pep–parent LL-34) at 2 μM for 18 h. IL-6 ELISA results are shown (n = 3). *Green bars* denote loss of IL-6 protein secretion compared with parent control shown in *red*. *C*, helical wheel plot for LL-34. Amino acids with *green circles* denote specific residues where replacement with an alanine caused the loss of IL-6 secretion compared with parent LL-34 peptide in NHEKs cotreated with U1 dsRNA. *D*, in the helical wheel plot for LL-34, *circled green* amino acids indicate that a substitution caused a loss of vetting in HDMEC. *E*–*H*, NHEKs were treated with LL-34-parent peptide (LL-34-P), LL-34-F5A, LL-34-I24A, LL-34-F27A, and LL-34-L31A at 2 μM, either individually or in combination with U1 dsRNA (2.5 μg/ml) for 8 h. Gene expression of IL-6, IFNB1, TSLP, and CXCL10 was assessed with qRT-PCR. GAPDH was used as a reference gene (n = 3) (∗*p* < 0.05; ∗∗∗∗*p* < 0.0001). One way-ANOVA was used. IL-6, interleukin 6; LL-34, 34-amino acid peptide; MIC, minimum inhibitory concentration, ns, not significant.

A synthetic peptide with a deletion of three amino acids at the C terminus of LL-37 (henceforth known as LL-34 parent) retains both its antibacterial activity against group A *Streptococcus* (GAS) and its ability to promote an inflammatory response in response to U1 dsRNA (30). Since LL-34 was the minimal truncation mutant of LL-37 that retained both antimicrobial and immune modulatory activity, we prepared a series of synthetic peptides with sequential substitutions of alanine at each amino acid position of the LL-34 parent peptide (Table 1). We compared the minimum inhibitory concentration (MIC) for the LL-34 parent peptide and the alanine mutants (LL-34 peptides—L1A and R34A) against methicillin-resistant *Staphylococcus aureus* strain USA300 (Fig. 1*A*). LL-34 peptides were incubated at concentrations of 0 to 100 μM with bacteria. The minimal concentration at which no growth was observed was noted after 18 h of incubation. LL-34—I13A, F17A, I24A, L28A, and L31A—peptides killed *S. aureus* USA300 more potently as compared with the LL-34 parent peptide that had an MIC of 25 μM, whereas LL-34—K12A, K35A, and R34S—mutants had a reduced MIC compared with the parent peptide. Because of these changes in the MIC against *S. aureus* between the mutants, we investigated the effective toxicity profile of the peptides in normal human epidermal keratinocytes (NHEKs) by lactate dehydrogenase (LDH) release assay (Table 1). The minimal concentration at which LL-34 parent peptide resulted in 50% LDH release by NHEKs was 5 μM. Compared with LL-34 parent peptide, G3A and I24A were more tolerated by the cells and had a minimal cytotoxic concentration of 20 μM. Most other peptides were cytotoxic at a minimal concentration of 10 μM, whereas LL-34—K15A was toxic at a concentration of 2.5 μM. Based on these results, we carried out all further experiments at 2 μM for LL-34 peptides to offset any effects observed because of toxicity.

**Table 1:** LL-34 parent peptide and its alanine scan mutants are shown

Peptide annotation	Peptide sequence	Minimal cytotoxic concentration in NHEK (μM)
LL-34—parent	LLGDFFRKSKEKIGKEFKRIVQRIKDFLRNLVPR	5
LL-34—L1A	ALGDFFRKSKEKIGKEFKRIVQRIKDFLRNLVPR	5
LL-34—L2A	LAGDFFRKSKEKIGKEFKRIVQRIKDFLRNLVPR	5
LL-34—G3A	LLADFFRKSKEKIGKEFKRIVQRIKDFLRNLVPR	20
LL-34—D4A	LLGAFFRKSKEKIGKEFKRIVQRIKDFLRNLVPR	5
LL-34—F5A	LLGDAFRKSKEKIGKEFKRIVQRIKDFLRNLVPR	10
LL-34—F6A	LLGDFARKSKEKIGKEFKRIVQRIKDFLRNLVPR	5
LL-34—R7A	LLGDFFAKSKEKIGKEFKRIVQRIKDFLRNLVPR	5
LL-34—K8A	LLGDFFRASKEKIGKEFKRIVQRIKDFLRNLVPR	5
LL-34—S9A	LLGDFFRKAKEKIGKEFKRIVQRIKDFLRNLVPR	5
LL-34—K10A	LLGDFFRKSAEKIGKEFKRIVQRIKDFLRNLVPR	5
LL-34—E11A	LLGDFFRKSKAKIGKEFKRIVQRIKDFLRNLVPR	5
LL-34—K12A	LLGDFFRKSKEAIGKEFKRIVQRIKDFLRNLVPR	5
LL-34—I13A	LLGDFFRKSKEKAGKEFKRIVQRIKDFLRNLVPR	5
LL-34—G14A	LLGDFFRKSKEKIAKEFKRIVQRIKDFLRNLVPR	5
LL-34—K15A	LLGDFFRKSKEKIGAEFKRIVQRIKDFLRNLVPR	2.5
LL-34—E16A	LLGDFFRKSKEKIGKAFKRIVQRIKDFLRNLVPR	5
LL-34—F17A	LLGDFFRKSKEKIGKEAKRIVQRIKDFLRNLVPR	5
LL-34—K18A	LLGDFFRKSKEKIGKEFARIVQRIKDFLRNLVPR	10
LL-34—R19A	LLGDFFRKSKEKIGKEFKAIVQRIKDFLRNLVPR	10
LL-34—I20A	LLGDFFRKSKEKIGKEFKRAVQRIKDFLRNLVPR	5
LL-34—V21A	LLGDFFRKSKEKIGKEFKRIAQRIKDFLRNLVPR	5
LL-34—Q22A	LLGDFFRKSKEKIGKEFKRIVARIKDFLRNLVPR	5
LL-34—R23A	LLGDFFRKSKEKIGKEFKRIVQAIKDFLRNLVPR	10
LL-34—I24A	LLGDFFRKSKEKIGKEFKRIVQRAKDFLRNLVPR	20
LL-34—K25A	LLGDFFRKSKEKIGKEFKRIVQRIADFLRNLVPR	10
LL-34—D26A	LLGDFFRKSKEKIGKEFKRIVQRIKAFLRNLVPR	10
LL-34—F27A	LLGDFFRKSKEKIGKEFKRIVQRIKDALRNLVPR	10
LL-34—L28A	LLGDFFRKSKEKIGKEFKRIVQRIKDFARNLVPR	5
LL-34—R29A	LLGDFFRKSKEKIGKEFKRIVQRIKDFLANLVPR	5
LL-34—N30A	LLGDFFRKSKEKIGKEFKRIVQRIKDFLRALVPR	10
LL-34—L31A	LLGDFFRKSKEKIGKEFKRIVQRIKDFLRNAVPR	10
LL-34—V32A	LLGDFFRKSKEKIGKEFKRIVQRIKDFLRNLAPR	10
LL-34—P33A	LLGDFFRKSKEKIGKEFKRIVQRIKDFLRNLVAR	10
LL-34—R34A	LLGDFFRKSKEKIGKEFKRIVQRIKDFLRNLVPA	10

The minimal cytotoxic concentration for each peptide (LD50) is shown.

We hypothesized that sequential replacement of each amino acid in LL-34 peptide with a neutral amino acid alanine might reveal the amino acid residues critical for its ability to induce inflammation in response to U1 dsRNA. First, we treated NHEKs and human dermal microvascular endothelial cells (HDMECs) with LL-34 parent peptide and each of the 34 mutants at 2 μM for 18 h and measured the secretion of interleukin 6 (IL-6) in the supernatant by ELISA (Fig. S1, *A* and *B*). Treatment with peptides alone did not significantly alter basal release of IL-6 in either NHEKs or HDMECs. We then treated both NHEKs and HDMECs with a combination of U1 dsRNA (2.5 μg/ml) and the LL-34 parent/mutant peptides at 2 μM for 18 h and measured IL-6 secretion (Fig. 1, *A*–*C*). NHEKs cotreated with U1 dsRNA and either of the LL-34 peptides—F5A, F6A, K12A, I13A, F17A, I20A, R23A, I24A, F27A, L28A, L31A, and V32A—showed reduced secretion of IL-6 protein as compared with cells treated with a combination of U1 dsRNA and LL-34 parent peptide (Fig. 1*A*). A similar pattern of IL-6 release was observed when HDMECs were treated with the peptide library and U1 dsRNA (Fig. 1*B*). Mapping each of the amino acids that were replaced with alanine leading to this loss of IL-6 secretion on a helical wheel plot (circled in *green*) showed that substitutions on the hydrophobic core resulted in this loss (Fig. 1, *C* and *D*). Also, tracking the mutations of the helical wheel plot similarly showed that mutations on the hydrophobic regions affected the ability of LL-34 peptides to vet U1 dsRNA (Fig. 1*D*).

We further looked at mRNA abundance by quantitative RT-PCR (qRT-PCR) to evaluate gene expression for type 1 interferon *IFNB1*, chemokine *CXCL10*, and cytokines *TSLP* and *IL6* in NHEKs treated with selected LL-34 parent, LL-34—F5A, I24A, F27A, and L31A—peptides either alone or in combination with U1 dsRNA for 18 h (Fig. 1, *E*–*H*). Treatment with peptides F5A, I24A, F27A, and L31A increased gene expression of TSLP, whereas the basal expression of *IL6*, *IFNB1*, and *CXCL10* was not affected. Cotreatment with U1 dsRNA showed a reduction in gene expression of *IL6*, *IFNB1*, and *CXCL10* for cells treated with F5A, I24A, F27A, and L31A. Comparison of antimicrobial action of these selected peptides on the growth of *S. aureus* strain USA300 and SA112, GAS, and *Escherichia coli* showed that peptides that lost the capacity to induce cytokine responses such as Pep F5A, I24A, F27A, and L31A had increased capacity to inhibit growth of most of the tested Gram-positive bacteria with little action against Gram-negative *E. coli* (Fig. S1, *C*–*F*).

Since LL-34 peptides—I24A and L31A—selectively demonstrated enhanced antimicrobial activity but loss of inflammatory activity with U1 dsRNA, we continued analysis with a focus on these peptide mutants to further understanding of the sequence dependence for inflammation.

### LL-34 mutants I24A and L31A lose capacity to promote inflammatory gene expression and recruit immune cells in mice

**Figure 2:**
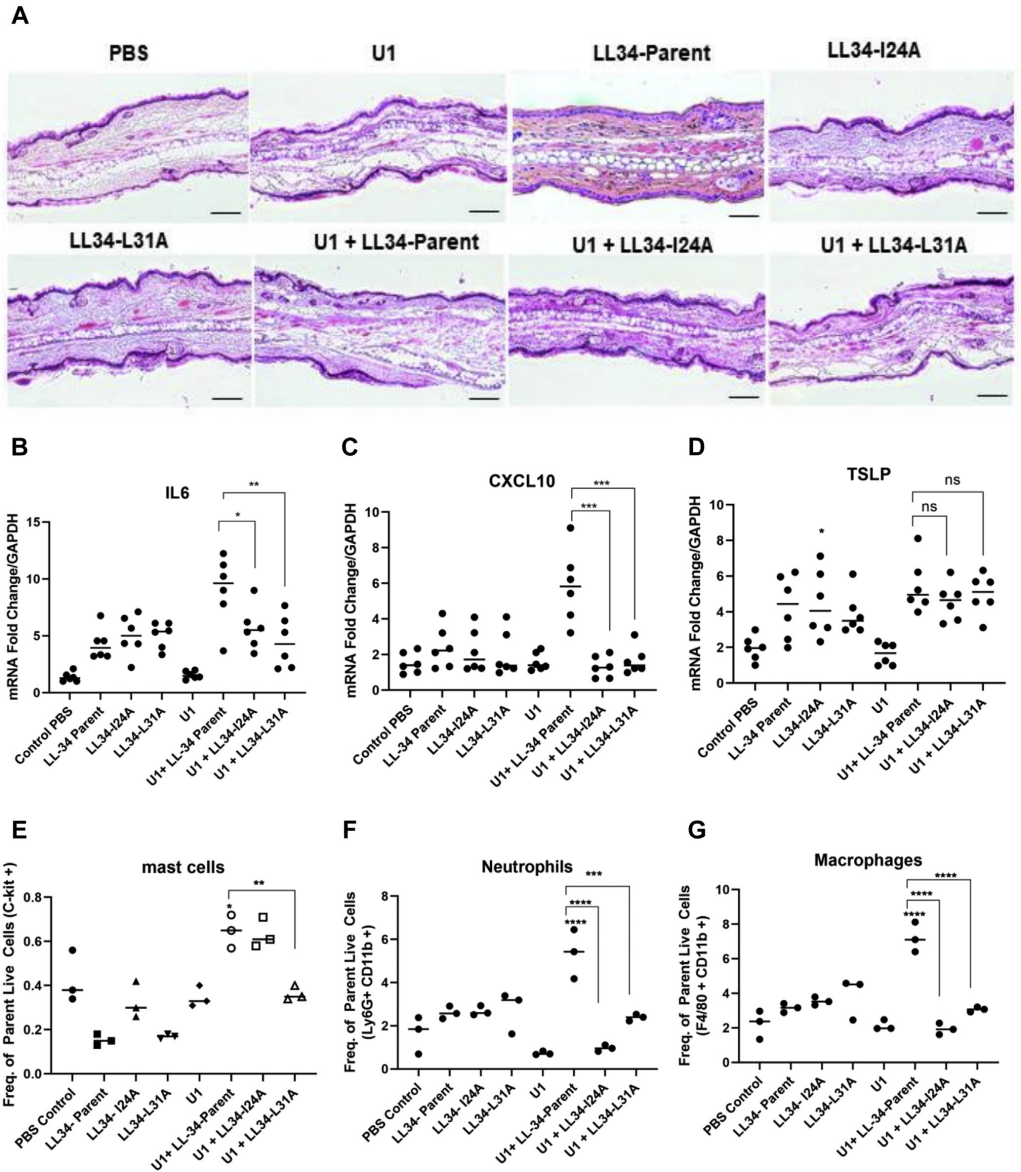
**LL-34-I24A and LL-34-L31A peptides loose vetting activity *in vivo*.** C57BL/6 mice ears were injected intradermally with either 25 μg of U1 dsRNA, 16 μM LL-34-parent, LL-34-I24A, and LL-34-L31A as shown in the figure for 24 h (n = 6). *A*, H&E staining of cross sections of mice injected with U1 dsRNA or LL-34 peptides is shown. The bar represents 20 μM. *B*–*D*, gene expression changes for *IL-6*, *CXCL10*, and *TSLP* were assessed with qRT-PCR. GAPDH was used as a reference gene. *E*–*G*, flow cytometry analysis was performed to assess the infiltration of immune cells in mouse ears injected with U1 dsRNA alone or a combination of U1 dsRNA and LL-34 peptides. Data are represented as the frequency of gated live cells for mast cells, macrophages, and neutrophils. (n = 3) (∗*p* < 0.05; ∗∗*p* < 0.01; ∗∗∗*p* < 0.001; ∗∗∗∗*p* < 0.0001). One way-ANOVA was used. LL-34, 34-amino acid peptide; ns, not significant.

C57BL/6 female mice at 8 weeks old were injected in the ears intradermally with a single injection of 25 μg of U1 dsRNA and/or LL-34 parent, Pep I24A, or Pep L31A. H&E staining of the cross sections are shown in Figure 2*A*. We investigated gene expression of *IL-6*, *CXCL10*, and *TSLP* in these mice (Fig. 2, *B*–*D*). The gene expression of *IL-6* (Fig. 2*B*) and *CXCL10* (Fig. 2*C*) was significantly reduced in mice coinjected with U1 dsRNA and either I24A and L31A mutant compared with ears injected with U1 dsRNA and LL-34 parent. This was in agreement with the *in vitro* results (Fig. 1). We also looked at the infiltration of immune cells to the mouse ear skin with flow cytometry. A notable increase in mast cells, macrophages, and neutrophils was observed in mice coinjected with U1 dsRNA and LL-34 parent peptide (Fig. 2, *E*–*G*). Coinjection with U1 dsRNA and I24A similarly increased mast cells in the skin, whereas a significant decrease in mast cell number was observed in ears coinjected with U1 dsRNA and L31A (Fig. 2*E*). Cotreatment with either U1 dsRNA and I24A or U1 dsRNA and L31A showed a significant reduction in macrophage and neutrophil recruitment compared with ears injected with LL-34 parent peptide and U1 dsRNA, indicating that the mutation caused a loss of innate vetting activity *in vivo* (Fig. 2, *F* and *G*). Overall, these data in mice confirm results seen in cell culture that LL-34 mutants I24A and L31A have decreased capacity to promote inflammation.

### Global transcriptional analysis reveals loss of type 1 interferon and cytokine signaling in LL-34 I24A and L31A mutants

**Figure 3:**
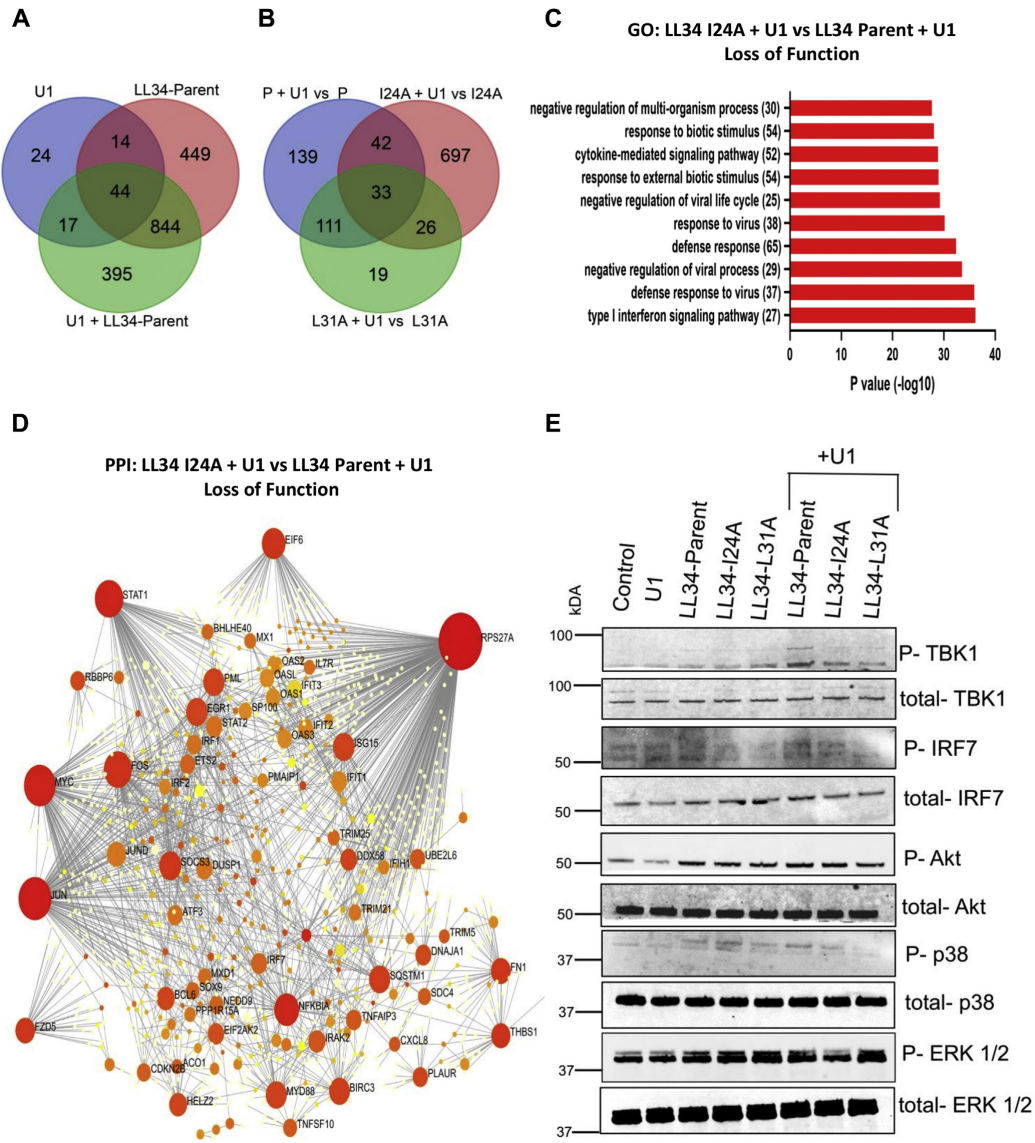
**LL-34 I24A and L31A mutations result in loss of innate immune vetting of U1 dsRNA leading to repressed activation of antiviral and innate immune pathways.***A*, Venn diagram for all differentially expressed genes from NHEKs treated with U1 dsRNA, LL-34-parent, and combinations of either U1 dsRNA and LL-34-parent. *B*, Venn diagram for showing genes differentially regulated in cells treated with U1 dsRNA and LL-34-I24A *versus* LL-34-I24A and U1 dsRNA and LL-34-L31A *versus* LL-34-L31A mutants in comparison to U1 dsRNA and LL-34-parent *versus* LL-34-parent as shown. *C*, gene ontology (GO) pathway analysis of genes downregulated in cells cotreated with U1 dsRNA and LL-34-I24A *versus* U1 dsRNA and LL-34-parent. *D*, protein–protein interaction networks were derived from cells cotreated with U1 dsRNA and LL-34 I24A mutant peptide and compared with cells treated with U1 dsRNA and LL-34 parent. *Red seeds* indicate genes that form over-represented networks and are indispensable. *E*, NHEKs were treated with U1 dsRNA (2.5 μg/ml) and/or LL-34-parent, LL-34-I24A, and LL-34-L31A (2 μM). Protein expression of p-TBK1, TBK1, p-IRF7, IRF7, p-Akt, Akt, p-ERK1/2, and ERK1/2 was assessed with immunoblot. ERK1/2, extracellular signal–regulated protein kinase; IRF7, interferon regulatory factor 7; LL-34, 34-amino acid peptide; NHEKs, normal human epidermal keratinocytes; TBK1, TANK-binding kinase 1.

RNA-Seq was utilized to obtain a global transcriptomic profile and map unique immune pathways in NHEKs exposed to U1 dsRNA, LL-34 parent, Pep I24A, Pep L31A, and a combination of U1 dsRNA and LL-34 parent, Pep I24A, or Pep L31A. Of all the differentially expressed genes (fold change of 1.5), Venn diagrams showed 395 unique genes in cells' cotreatment with U1 and LL-34 parent. Similarly, 448 and 325 unique differentially regulated genes were observed after U1 + I24A and U1 + L31A treatments, respectively (Fig. 3, *A* and *B* and Fig. S2, *A* and *B*). Gene Ontology analysis showed enrichment of several pathways that were not induced in cells treated with U1 dsRNA and I24A compared with U1 dsRNA and LL-34 parent peptide treatment (Fig. 3*C*). These pathways included type 1 interferon signaling, cytokine signaling, response to external biotic stimulus, and cytokine-mediated signaling pathway. Reconstruction of protein–protein interaction networks from genes affected upon cotreatment with U1 and I24A in comparison to U1 and LL-34 parent showed that type I interferon signaling pathway–regulated genes *STAT*, *ISG15*, *OAS1*, and *IRF2*, and retinoic acid–inducible gene I (RIGI) antiviral pathway genes, *DDX58* and *MAVS*, were repressed (Fig. 3*D*). We verified the repression of mitochondrial antiviral signaling (MAVS) with immunoblot analysis. NHEKs were treated with U1 dsRNA, LL-34 parent, Pep I24A, Pep L31A, or with a combination of U1 and LL-34 parent, U1 and Pep I24A, or U1 and Pep L31A (Fig. 3*E*). Decreased phosphorylation of TANK-binding kinase 1 (TBK1) and interferon regulatory factor 7 (IRF7) was observed in cells cotreated with U1 and I2A/L31A in comparison to U1 and LL-34 parent peptide treatment.

Overall, the RNA-Seq data that showed substitutions with alanine at I24 and L31 positions caused the loss of antiviral and innate immune signaling in keratinocytes exposed to U1 dsRNA and validated prior *in vitro* and *in vivo* observations made on candidate genes.

### Substitution at LL-34 positions 24 and 31 eliminate binding of U1 to cell surface scavenger receptors

**Figure 4:**
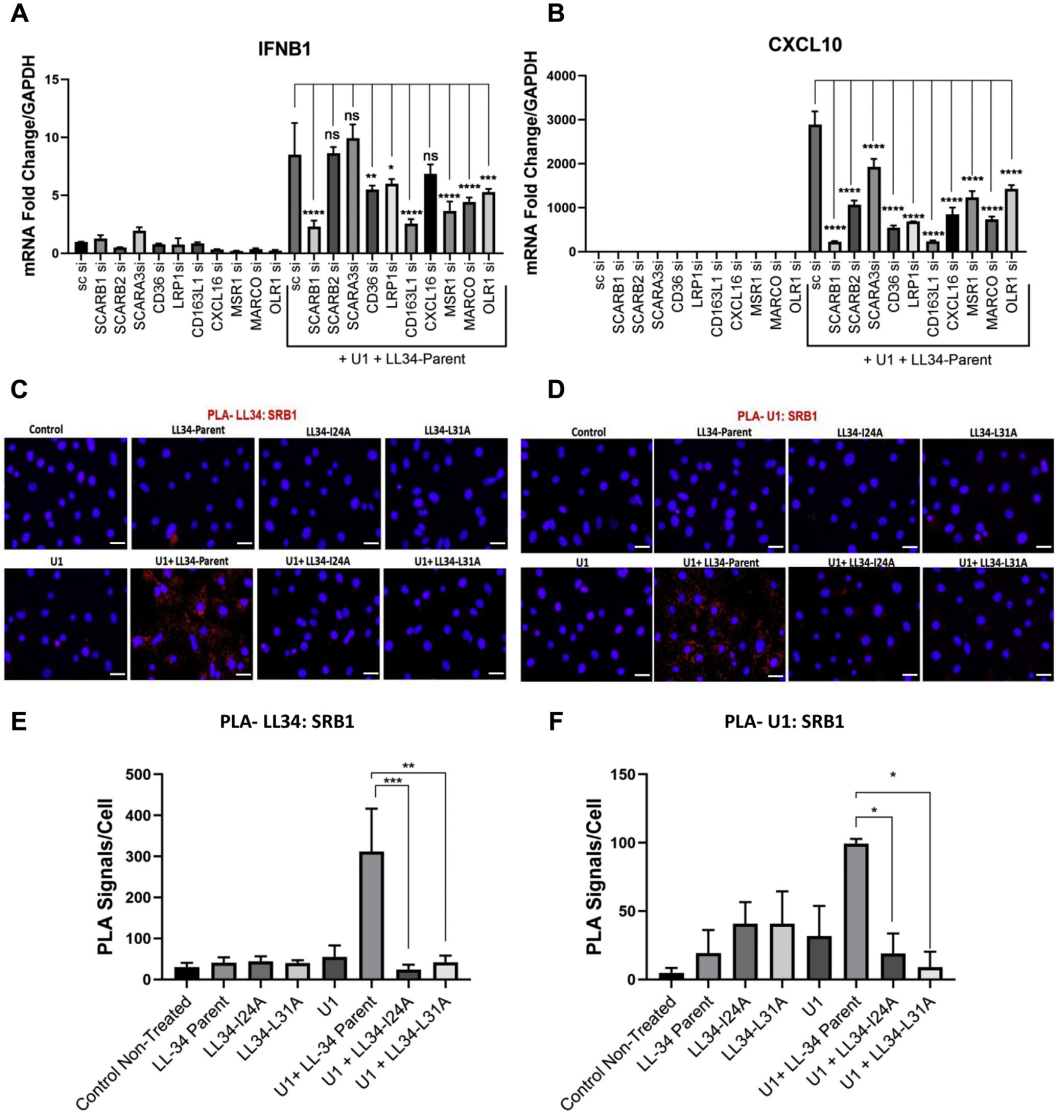
**LL-34 enables U1 dsRNA association with scavenger receptor (SR) SRB1.***A* and *B*, NHEKs with gene knockdown for SRs SCARB1, SCARB1, SCARA3, CD36, LRP1, CD163L1, CXCL16, MSR1, MARCO, and OLR1 were cotreated with U1 dsRNA (2.5 μg/ml) and LL-34 (2 μM) for 18 h. Gene expression of IFNB1 and CXCL10 was assessed with quantitative RT-PCR (n = 3). *C*, proximity ligation assay (PLA) for U1 dsRNA and SR SRB1 binding. NHEKs were treated with LL-34-parent, LL-34-I24A, and LL-34-L31A (2 μM) for 15 min, followed by treatment with biotinylated U1 dsRNA (2 μg/ml) for 60 min at 4 °C. The physical proximity of LL-34 and SRB1 (*C*), and U1 dsRNA, and SRB1 (*D*) was determined with fluorescence-based PLA that produces a red fluorescent signal. DAPI (*blue*) was used as a nuclear counterstain. The scale bar represents 50 μm. Signal counts of *C* and *D* from five visual fields were used for PLA signal quantification, as shown in *E* and *F* (n = 3). (∗*p* < 0.05; ∗∗*p* < 0.01; ∗∗∗*p* < 0.001; ∗∗∗∗*p* < 0.0001). One-way ANOVA was used. DAPI, 4′,6-diamidino-2-phenylindole; LL-34, 34-amino acid peptide; NHEKs, normal human epidermal keratinocytes; ns, not significant.

In previous studies, we have shown that the complex of U1 RNA and LL-37 is recognized by cell surface scavenger receptors (SRs) prior to internalization. Upon internalization *via* the SRs, the U1 RNA–LL-37 complex is recognized by either the cytoplasmic RIGI/MAVS or the endosomal TLR3 pattern recognition receptors to initiate antiviral and proinflammatory signaling (7, 30). Hence, we next looked at which SRs are most critical to the activity of the parent LL-34 peptide. NHEK cells were transfected with siRNAs against SR genes *SCARB1* (encoding SRB1), *SCARB2*, *SCARA3*, *CD36*, *LRP1*, *CD163L1*, *CXCL16*, *MSR1*, *MARCO*, and *OLR1*. Knockdown of SRs was confirmed with qRT-PCR. A scrambled nontargeting siRNA was used as a control (Fig. S3). NHEKs were then cotreated with U1 dsRNA (2.5 μg/ml) and LL-34 parent peptide (2 μM) for 18 h in cells after SR knockdown (Fig. 4, *A* and *B*). A knockdown of multiple individual SRs showed reduced expression of IFNB1 or CXCL10 when compared with U1 and LL-34 parent (Fig. 4, *A* and *B*). This is in line with previous reports that suggest that multiple SRs may form a complex with U1 dsRNA and LL-37 peptide (30).

Since cytokine responses to LL-34 showed the greatest dependence on the expression of the SR SCARB1 (SRB1) in NHEK, we next looked at whether I24 and L31 peptides influence formation of a complex with U1 RNA. The binding of peptide to SRB1 or U1 to SRB1 was assessed by proximity ligation assay (PLA) (Fig. 4, *C*–*E*). Analysis of NHEKs by PLA showed that the presence of LL-34 parent enabled LL-34 protein to localize with SRB1, whereas Pep I24A and Pep L31A did not achieve close localization (spatial correlation <40 nm) (Fig. 4, *C* and *E*). Similarly, binding of U1 dsRNA to SRB1 was seen with parent LL-34 but not with either I24A or L31A (Fig. 4, *D* and *F*).

**Figure 5:**
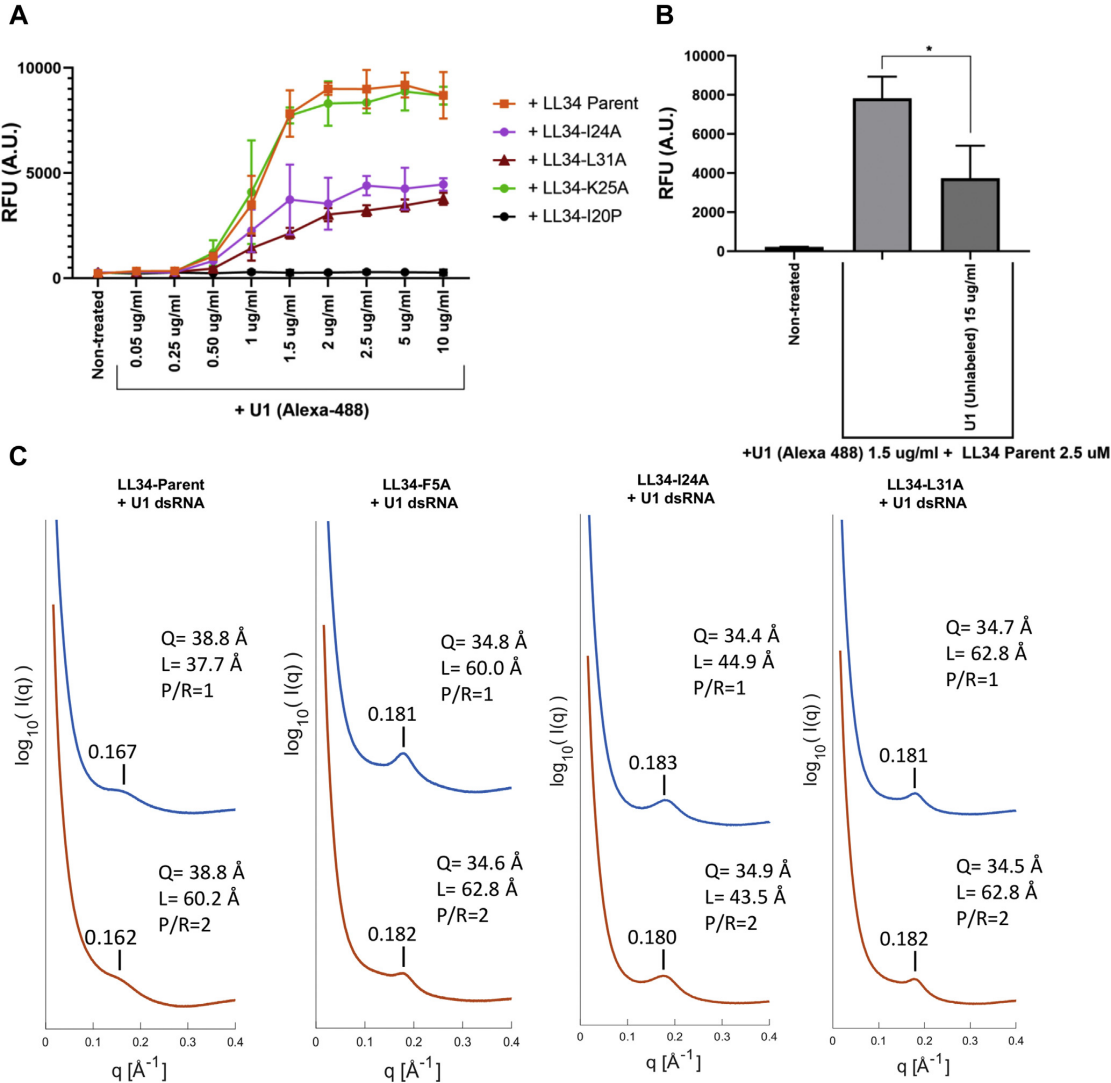
**Biophysical properties of binding of U1 dsRNA to LL-34 peptides and SRB1 protein.***A*, NHEKs were treated with Alexa 488–labeled U1 dsRNA or a combination of Alexa 488–labeled U1 dsRNA (0.05–10 μg/ml) and LL-34 peptides LL-34 parent, LL-34 I24A, LL-34 K25A, LL-34 L31A, and LL-34 I20P at 2.5 μM as shown. The cells were prechilled on ice for 15 min before the addition of U1 dsRNA and/or LL-34 peptides, and the surface binding of U1 dsRNA was allowed for 10 min. The amount of U1 dsRNA binding to NHEKs was assessed with a fluorometer, and data are represented as relative fluorescence units (RFUs) (n = 3). *B*, NHEKs were similarly treated with Alexa-labeled U1 dsRNA (1.5 μg/ml) and/or unlabeled U1 dsRNA (15 μg/ml) with the addition of LL-34 parent peptide at 2.5 μM. The experiment was performed on ice for 10 min, and the relative binding of Alexa 488 U1 dsRNA was assessed on a fluorometer and is shown as RFU (n = 3) (∗*p* < 0.05) one-way ANOVA. *C*, SAXS spectra of LL-34 peptides LL-34 parent, LL-34 F5A, LL-34 I24A, and LL-34 L31A incubated with U1 dsRNA. The peptide-U1 dsRNA (P/R) ratio is varied at 1 and 2 for all conditions. The U1 dsRNA interhelical spacing (Q) was calculated by the peak position. The domain size (L) was calculated by the full width at half maximum of the first peak. LL-34, 34-amino acid peptide; NHEKs, normal human epidermal keratinocytes; SAXS, small-angle X-ray scattering.

We also assayed the dynamics of binding of U1 dsRNA on the surface of NHEKs in the presence of LL-34 mutant peptides. NHEKs were exposed to fluorescent Alexa 488–labeled U1 at increasing concentrations (0.05–10 μg/ml) and LL-34 parent peptide, LL-34 I24A, LL-34 L31A, LL-34 K25A, and LL-34 proline at isoleucine position 20 (I20P) for 10 min on ice to allow for surface binding of U1 to cells in the presence of peptides (Fig. 5*A*). LL-34 parent peptide and LL-34 K25A caused enhanced binding of U1 on the cell surface, compared with LL-34 I24A and LL-34 L31A. Treatment with an LL-34 variant that substituted I20P showed no binding of U1 dsRNA on NHEKs. The addition of excess unlabeled U1 dsRNA (15 μg/ml) in the presence of Alexa 488 U1 RNA (1.5 μg/ml) and LL-34 parent peptide inhibited the binding of Alexa 488 U1 dsRNA to NHEKs, compared with control and to a level of association similar to the nonsaturable binding observed for I24A and L31A (Fig. 5*B*). These observations suggest that while some association with the cell occurs for U1 RNA, receptor-specific saturable binding is dependent on LL-34 and disrupted by amino acid substitutions similar to those that inhibit capacity to promote inflammation or bind U1 to SRs.

Taken together, we conclude that LL-34 amino acid substitutions resulting in the inability of peptides to promote inflammation by immune vetting of dsRNA also result in an inability to enable binding of U1 RNA to SRs and subsequent reduced binding to the cell surface.

### Complexes between U1 and LL-34 parent or mutant peptides exhibit cognate supramolecular structures that permit binding to TLR3

The structures of U1 dsRNA–LL-34 peptide complexes were next studied by high-resolution synchrotron small-angle X-ray scattering (SAXS) to compare with prior structural assessments required for association of U1 with TLR3. LL-34 parent peptide and three mutant peptides, LL-34 F5A, LL-34 I24A, and LL-34 L31A, were individually incubated with U1 dsRNA to form complexes at two specific peptide-to-dsRNA molar ratios (P/R = 1, 2). SAXS measurements show an unambiguous diffraction peak at Q = 0.167 Å^−1^ for complexes between the LL-34 parent peptide and U1 dsRNA (Fig. 5*C*). This diffraction pattern is consistent with the formation of a supramolecular complex with an effective dsRNA packing state at an average interhelical spacing of 38.8 Å. Moreover, as we altered the stoichiometry in favor of the LL-34 (P/R = 2), the basic structure was preserved. This basic diffraction signature was also seen in U1 complexes made with other LL-34 mutant peptides. They all exhibited a similar inter-dsRNA spacing of ∼35 Å (Fig. 5*C*). From previous work, we expect complexes with inter-RNA spacing in this range (34 –39 Å) to strongly activate TLR3. Interestingly, although the interhelical spacing was not influenced by the alanine mutation, at P/R ratio 1, the domain size for LL-34 I24A and LL-34 L31A increased compared with LL-34 parent peptide, which can impact the degree of amplification (Fig. 5*C*). Since all these LL-34-U1 dsRNA complexes exhibit structures that can in principle amplify TLR3 activation, the observed differential immune activation is all the more striking and suggests that immune vetting of these complexes at the cell entry stage plays a pivotal role.

## Discussion

A study of the sequence–function relationship by alternations in amino acid residues by either truncations or substitutions has revealed the residues critical for the antimicrobial and inflammatory response of cathelicidin (13). In this study, we further studied the effect of sequential substitution of LL-34 peptide with alanine on the antibacterial and proinflammatory activity.

In our prior study, we developed several truncation mutants of LL-37 peptide and showed that a 34-amino acid sequence peptide LL-34 derived from LL-37 retained both the antibacterial and immunomodulatory capacity (30). Specifically, by substitution of isoleucine with proline, we showed that the modified LL-34 I20P peptide retained its antimicrobial activity against GAS; however, it lost the capacity to recognize U1 dsRNA released from UV-damaged cells, and hence failed to initiate an inflammatory response in the psoriatic skin. Thus, we previously concluded that the antimicrobial activity of cathelicidin may be distinct from its ability to recognize U1 dsRNA. To more closely study this phenomenon of innate immune vetting of damaged RNA by cathelicidin, we generated a library of alanine substitution mutants (Table 1). Alanine scanning strategy was used since single substitutions with alanine does not cause steric or electrostatic changes in the peptide and also does not alter the main-chain confirmation like the proline or glycine substitutions (31, 32). Treatment of keratinocytes and human dermal endothelial cells by all the 34 mutants showed minor differences in the cytotoxicity profile, and no change in the secretion of the proinflammatory chemokine IL-6 was observed (Fig. S1). Since it has been previously shown that alanine and lysine substitutions in cathelicidin-derived peptide KR-12 may enhance or inhibit its antibacterial response (33), we tested the activity of all the 34 mutants against *S. aureus* USA300 and noticed that LL-34 peptides—IL3a, F17A, I24A, and L31A—gained antimicrobial activity (Fig. 1). We further investigated the ability of the mutants to recognize U1 dsRNA (Fig. 1). Both I24A and L31A mutant treatment showed reduced IL-6 protein secretion compared with the parent LL-34 peptide when the cells were treated in combination with U1 dsRNA, indicating a possible loss of ability to recognize U1 dsRNA. The I24A and L31A mutants were both immunologically active as increased gene expression of TSLP chemokine was seen.

We had previously shown that cotreatment with U1 dsRNA and LL-37 generates a strong inflammatory response in mice when injected intradermally in the ears (7). We noticed a similarly strong response in mice upon treatment with a combination of U1 dsRNA and LL-34 peptide, with enhanced IL-6 and CXCL10 proinflammatory cytokine expression and increased infiltration of immune cells to the mouse ears. Coinjection of I24A or L31A and U1 dsRNA showed a diminished immune response indicating a loss of recognition of RNA because of the alanine mutation (Fig. 2). To gain a deep understanding of the downstream pathways affected by the alanine mutation, we obtained a global transcriptomic profile of keratinocytes treated with LL-34 parent peptide, I24A, L31A in combination with U1 dsRNA. GO analysis showed that treatment with mutant peptides in combination with U1 dsRNA resulted in suppression of pathways related to antiviral type 1 interferon signaling and inflammatory cytokine response (Fig. 3). In inflamed psoriatic and rosacea skin, the RIGI/MAVS signaling pathway is activated upon recognition of the self nucleic acids by cathelicidin, which leads to activation of a downstream type 1 interferon response and adaptive priming to dendritic cell–mediated T cell activation (7, 9). Our data showed that the I24A and L31 mutations caused a loss of the RIGI/MAVS signaling indicated by the reduced phosphorylation of TBK1 and IRF7 (Fig. 3).

The double-stranded viral RNA mimic poly IC has been shown to activate a proinflammatory type 1 interferon response by engaging cell surface SRs and initiated the inflammatory response by delivery of the poly IC to the cytosol or endosome and activation of RIG1 or TLR3 pattern recognition receptors (34, 35, 36). An important distinction we observed in our previous study is that unlike poly IC, self RNA U1 is inert and not recognized by the cells unless complexed by LL-37 (9, 30). The coupling of U1 to LL-37 results in the engagement of multiple SRs on the cell surface leading to cellular uptake *via* clathrin-mediated endocytosis. The concept of vetting by other LL-37 like positively charged proteins has been also noted. In macrophages, curli amyloid fibrils associate with DNA from damaged cells and activate intracellular TLR9 *via* toll-like receptor 3–mediated uptake, initiating an inflammatory response in Alzheimer's disease (37). A recent study also showed that a type 1 interferon–regulated chemokine CXCL10 is released from plasmacytoid dendritic cells during skin wounding and recognizes commensal bacterial nucleic acids to initiate a protective inflammatory response to initiate wound closure by enhancing the production of growth factors like epidermal growth factor (38). Thus, the initiation of innate immune vetting by alarmins like LL-37 and others may serve as both a protective and detrimental immune response depending on the context of vetting initiation by danger-associated molecular patterns.

The uptake of nucleic acid–LL-37 peptide complexes is dependent on SRs. SRs constitute a unique class of pattern recognition receptors that recognize and bind several danger-associated molecular patterns, including whole microorganisms or subparts like lipoteichoic acids, modified lipids like oxidized low-density lipoproteins, and can alter the immune cytokine responses in several diseases like atherosclerosis and Alzheimer's disease (39, 40). SRs constitute several subclasses that are structurally heterogeneous (39). Our previous study showed that although SRs are structurally distinct, several different SRs are cooperatively recruited to form complexes with U1 dsRNA and LL-37 peptide and have redundant functions that allow uptake *via* endocytosis (30). In this study, the knockdown of several SRs in keratinocytes differentially affected the expression of IL-6 and IFNB1 in cells treated with a combination of U1 dsRNA and LL-34 parent peptide (Fig. 4). With PLA, we show that the association of U1 with SR SRB1 is hindered when LL-34 is mutated at I24A and L31A positions, thus leading to a loss of downstream vetting response (Figs. 1 and 2). The SAXS data show unambiguously the electrostatically binding and mutual organization between LL-34 peptides and U1 dsRNA. Interestingly, the U1 dsRNA-binding ability is not inhibited, and the structures of peptide–U1 dsRNA complexes are not significantly altered by the alanine mutation sites at 5, 24, and 31. For LL-34 parent peptide and all these mutant peptides, the interhelical spacing of U1 dsRNA is corresponding to 34 to 39 Å. Previous study showed that inter-dsRNA spacings at 33 to 37 Å range has the highest activation correlate with TLR3 (41). Our results further indicated that the complexes of peptide and nucleic acid might be good candidates to be recognized by SR SRB1 and regulate proinflammatory activity; however, a defect in SRB1 recognition was noted in the I24A and L31A mutants indicating that the mutations affected confirmation to present dsRNA–peptide complex to SRs. Further studies are needed to elucidate how the I24A and L31A mutation affects SRB1-mediated uptake of the dsRNA–peptide complex and downstream presentation to pattern recognition receptors like RIGI and TLR3. Also, the stoichiometry of these interactions, capacity of the peptides to assemble or bind alone, and capacity of other molecules in addition to U1 RNA need to be evaluated. Targeting the binding of dsRNA–peptide complex may be a future therapeutic strategy. A recent study showed that spherical nucleic acid nanoparticles are readily uptaken by the epidermal barrier by recruitment of class A SRs and endocytosis *via* lipid rafts. This uptake can be effectively blocked by endocytic inhibitors (42).

In summary, our findings show that the hydrophobic face of the cathelicidin peptide is essential for its ability to recognize dsRNA and initiate an immune response. The intricate details of how the structure of cathelicidin contributes to the inflammatory activity will help the design of molecules that may act as competitors of cathelicidin, thus preventing inflammation and disease progression.

## Experimental procedures

### Cell culture and reagents

HDMECs (PromoCell) and NHEKs (Life Technologies) were cultured according to manufacturer's instructions in a CO_2_ incubator. LL-37 (Genemed) and LL-34 Alanine Scan Peptides (LifeTein) were reconstituted according to the manufacturer's instructions. The LL-34 alanine screen peptides were purified, desalted, and TLC removal was performed by the manufacturer. All the peptides were between 70 and 90% purity. Synthetic U1 dsRNA and its biotinylated form were synthesized as described previously (30). U1 dsRNA is a 164 bp spliceosome stem loop structure as described previously (8, 43). Similar to the biotinylated form, the Alexa 488 U1 RNA was synthesized and purified according to manufacturer's instructions (Lucigen).

### Mice

All mouse procedures were approved by the University of California, San Diego (UCSD) Institutional Animal Care and Use Program (protocol number: S09074). Eight-week-old female C57BL/6 wildtype mice were used for experiments. C57BL/6 mice ears were injected intradermally with U1 dsRNA and/or LL-34 peptides as described previously (7). Single injections were performed. A total of 30 μl was injected in the middle part of the ear. Twenty-four hours after the injection, mice were euthanized with CO_2_ asphyxiation and ears excised and immediately collected in cold RPMI complete medium on ice and processed for immunohistochemistry, gene expression analysis, and flow cytometry within 1 h.

### Flow cytometry

Mouse ears were collected following euthanasia in cold complete RPMI media on ice and cut into small pieces within 60 min, then digested with 2.5 mg/ml collagenase D, and 30 ng/ml DNase1 for 2 h at 37 °C and then filtered through a 30 μm filter to generate single-cell suspension for fluorescence-activated cell sorting analyses. Cells were then stained with Fixable Viability Dye eFluor 506 (eBioscience; 65-0866-14) blocked with antimouse CD16/32 (eBioscience; 14016185, 1:50 dilution), followed by staining with antibody cocktails for immune cells. The antibody cocktail for immune cells included PECy7-CD11b (BioLegend; 101216, 1:200 dilution), FITC-Ly6G (eBioscience; 11593182, 1:150 dilution), PE-F4/80 (eBioscience; 12480182, 1:200 dilution), APC-CD11C (BioLegend; 117310, 1:100 dilution), AF700-MHCII (eBioscience; 56532182, 1:100 dilution), and c-Kit (Biolegend; 10581, 1:100 dilution). Fluorescence-activated cell sorting analyses for surface expression of immune cell markers were performed by the Biorad ZE5 machine and analyzed by FlowJo V10 software (BD Biosciences). Dead cells were gated out from the analyses.

### ELISA

Cell culture supernatant was frozen at −80 °C until use for analysis. ELISA was performed with sandwich ELISA kits (R&D Systems) and quantified on a Spectramax Absorbance reader (Molecular Biosystems). Data were analyzed with GraphPad.

### *In vitro* antibacterial assay and MIC determination

Methicillin-resistant *S. aureus* USA300 and methicillin-sensitive *S. aureus* SA113 (American Type Culture Collection 35556) were cultured in 3% tryptic soy broth overnight at 37 °C with shaking at 250 rpm. GAS M49 strain NZ131 was cultured in Todd Hewitt broth supplemented with 1% yeast extract overnight at 37 °C without shaking. *E. coli* R1 strain RS218 was cultured in LB broth overnight at 37 °C with shaking at 250 rpm. MIC values were determined using a round-bottom 96-well microdilution method with RPMI cell culture media (as a source of Ca^2+^ and Mg^2+^) supplemented with tryptic soy broth, Todd Hewitt broth/yeast extract, or LB to facilitate growth of the respective bacterial strains. Overnight bacterial cultures were subcultured into fresh broth and grown to midlate log phase indicated by an absorbance at 600 nm value between 0.8 and 1.0 for each bacterial strain. The absorbance was then normalized to provide a concentration of 1 × 10^7^ colony-forming units/ml in RPMI medium. The LL-34 parent and alanine scan mutants were serially diluted into the 96-well microtiter plates containing prewarmed RPMI bacterial growth medium. A 10 μl aliquot of the midlate log phase bacteria was inoculated into the 96-well microtiter plates that contained 95 μl of RPMI medium with or without the LL-34 peptide dilutions and incubated for 16 to 18 h at 30 °C with shaking at 200 rpm. Inhibition of growth was determined by measuring the absorbance at 600 nm readings of each well using a microplate reader (Infinite M200 Pro; Tecan). The MIC of each bacterial strain was determined by the lowest peptide concentration that inhibited more than 80% bacterial growth.

### LDH assay

LDH cytotoxicity assay was performed according to manufacturer's instructions (Thermo Fisher Scientific). Culture supernatants from keratinocytes were used fresh for LDH assay after experimental treatments.

### RNA-Seq

RNA was extracted using a PureLink RNA Mini Kit (Life Technologies). Isolated RNA was submitted to the UCSD IGM Genomics Center for RNA-Seq performed on a high-output run V4 platform (Illumina) with a single-read 100 cycle runs. Data alignment was done on Partek Flow software (Partek) with Tophat2 (version 2.0.8). Gene Ontology enrichment analysis was performed on differentially regulated genes (≥1.5-fold) using Partek Flow software. The protein–protein interaction network was generated in the InnateDB database (44).

### Real-time qRT-PCR

RNA extracted from cells (Pure Link RNA Isolation Kit; Life Technologies) was quantified on the Nanodrop 2000/200C Spectrophotometer (Thermo Fisher Scientific). About 500 ng of purified RNA was used to synthesize complementary DNA with iScript cDNA Synthesis Kit (Bio-Rad). Predeveloped Taqman (Thermo Fisher Scientific) gene expression assays were used to evaluate mRNA transcript levels. Taqman cycling conditions were used as previously described (9, 45). All experiments were performed on a CFX96 system (Bio-Rad). Fold change relative to *GAPDH* was calculated using the Livak ΔΔC_t_ method (46).

### Immunoblot

Cells were lysed in complete radioimmunoprecipitation assay buffer with added 1× protease and Phosphatase Inhibitor Cocktail (Life Technologies). The lysate was centrifuged at 4 °C, 13,000 rpm for 20 min, and total cytoplasmic supernatant fraction was stored at −80 °C until future use. For mouse ear, whole immunoblot tissue was homogenized in 1× complete radioimmunoprecipitation assay buffer. Total protein was quantified for each treatment with Bio-Rad Protein Assay (Bio-Rad). About 10 μg of total protein was loaded onto a 4 to 20% Mini-PROTEAN TGX gel (Bio-Rad), transferred to a polyvinylidene difluoride membrane, and probed with primary antibodies. The following primary antibodies from Cell Signaling were used: p-TBK1 (5483; 1:1000 dilution), TBK1 (38066; 1:1000 dilution), p-IRF7 (5184; 1:1000 dilution), IRF7 (4920; 1:1000 dilution), p-Akt (4060; 1:1500 dilution), Akt (4685; 1:1500 dilution), p-p38 (4511; 1:1500 dilution), p38 (8690; 1:1000 dilution), phosphorylated extracellular signal–regulated protein kinase 1/2 (4370; 1:2000 dilution), and extracellular signal–regulated protein kinase 1/2 (4695; 1:2000 dilution). IRDye-conjugated anti-rabbit and antimouse secondary antibodies (IRDye800CW; Licor; 1:10,000 dilution) were used. The images were acquired on an Odyssey CLx Imaging System (Licor).

### Immunohistochemistry

Immunohistochemistry was performed as previously described (30, 45). Whole skin was fixed in 10% formaldehyde and preserved in 70% ethanol. Tissue sections were cut at the histopathology core facility at the UCSD Moores Cancer Center, and H&E staining of the ear sections (10 μM) were also acquired from the core. H&E images were acquired on Olympus BX41 microscope (widefield) with 10× objective.

### PLA

PLA was performed as described previously (30). NHEKs were cultured in 8-well chamber slides to 80% confluence and treated with LL-34 peptides for 15 min, followed by incubation with biotinylated U1 dsRNA (2 μg/ml) at 4 °C for 1 h. Unbound biotinylated U1 dsRNA and LL-34 peptides were removed with cold 1× PBS washes. Cells were then fixed in 4% paraformaldehyde in 1× PBS at 4 °C. Following fixation, cells were blocked for 60 min at 37 °C in a humidified chamber and incubated with antibiotin antibody (ab201341; Abcam; 1:100 in blocking buffer) and SRB1 antibody (NB400-104; Novus Biologicals; 1:100 dilution) for 60 min at room temperature. For LL-34 PLA binding, LL-37 antibody (sc-166770; Santa Cruz Biotechnologies, 1:1000 dilution) was used. After washing with 1× PBS, the cells were further incubated with Plus and Minus oligonucleotide probe–conjugated secondary antibodies to biotin (1:1000 dilution) and CD36 (1:1000 dilution) for 60 min at 37 °C in a humidified chamber. Further hybridization, ligation, amplification, and detection of the PLA was performed according to the manufacturer's instructions (Duolink, catalog no.: DUO92101, Sigma–Aldrich). PLA signals were captured on Olympus BX41 microscope (widefield) with 40× objective and counted by Duolink ImageTool software, version 1.0 (Sigma–Aldrich). Five fields were captured per treatment for quantification of PLA signals with ImageTool software. The experiment was repeated three independent times, and the immunofluorescence images are representative of a single experimental replicate.

### siRNA transfection

siRNAs against SRs were purchased from Thermo Fisher Scientific (Smartpool OnTarget Plus). siRNA transfection was performed as previously described for NHEKs (30).

### SAXS and data analysis

Structures of LL-34–U1 complexes were determined by synchrotron-based SAXS. Peptides (10 mg/ml) and U1 dsRNA were mixed at specific peptide-to-dsRNA molar ratios (P/R) and equilibrated overnight at room temperature. Precipitated peptide–RNA complexes were transferred into 1.5 mm quartz capillaries (Hilgenberg GmbH, Mark-tubes) and hermetically sealed. SAXS experiments were conducted at the Stanford Synchrotron Radiation Lightsource (9 keV) at BL 4-2 and the Advanced Light Source (10 keV) at BL 7.3.3. Samples were incubated at 37 °C and centrifuged before measurement. Scattered radiation was collected using a DECTRIS PILATUS3 X 1M detector (pixel size of 172 μm). The 2D powder diffraction patterns were azimuthally integrated into 1D patterns using the Nika 1.76 package for Igor Pro 7.04 (Wavemetrics) (47). For all samples, multiple measurements were taken at different times to ensure consistency.

To determine the structures, present in each sample, the integrated scattering intensity I(*q*) *versus q* was plotted using MATLAB. The lattice parameter d indicates the inter-dsDNA spacing between dsDNA columns. The inter-dsRNA spacing is estimated from the first peak position by the formula *Q* = 2π/*q*_1_. We also measured average domain size *L* of each sample. We approximated the structure factor peaks as squared-Lorentzian functions

$$S\left(q\right)=h^{3}/4\pi {\left({\left|q-q_{1}\right|}^{2}+{\left(h/2\right)}^{2}\right)}^{2}$$

where *q*_1_ is the location of the first peak, and *h* is the peak width (48). The experimental SAXS data were background subtracted, and the first peak for each complex was fitted using nonlinear least-squares regression in MATLAB. The diffraction pattern for disordered anisotropic systems can be complex, but the disordered bundles were considered as follows. There is only one single broad diffraction feature in *S*(*q*) suggests a disordered system with short-ranged exponential decay of positional correlations. Given *S*(*q*) has a Lorentzian form, averaging over all solid angles, we approximate the domain size as: $L=h^{\frac{1}{2}}/\left(h/2\right)$ (49).

### Statistical analysis

Normally distributed results are represented as means and standard deviation of the means. For comparison of multiple groups with ANOVA (Tukey post hoc test) was used, as indicated in legends to the figures. All the statistical analyses were performed on Prism 8 (GraphPad).

## Data availability

Datasets related to this article can be found at https://www.ncbi.nlm.nih.gov/geo/. The accession number of the article is GSE168050.

## Supporting information

This article contains supporting information.

## Conflict of interest

R. L. G. is a cofounder, scientific advisor, consultant, and has equity in MatriSys Biosciences and is a consultant, receives income, and has equity in Sente, Inc.
