# Supplementary material for: Sequence determinants in the cathelicidin LL-37 that promote inflammation via presentation of RNA to scavenger receptors
Source: J Biol Chem. 2021 May 26;297(1):100828. doi: 10.1016/j.jbc.2021.100828 (PMC8214221; doi:10.1016/j.jbc.2021.100828)
Supplement: Supplemental Figures S1–S3 [file mmc1.docx]

####
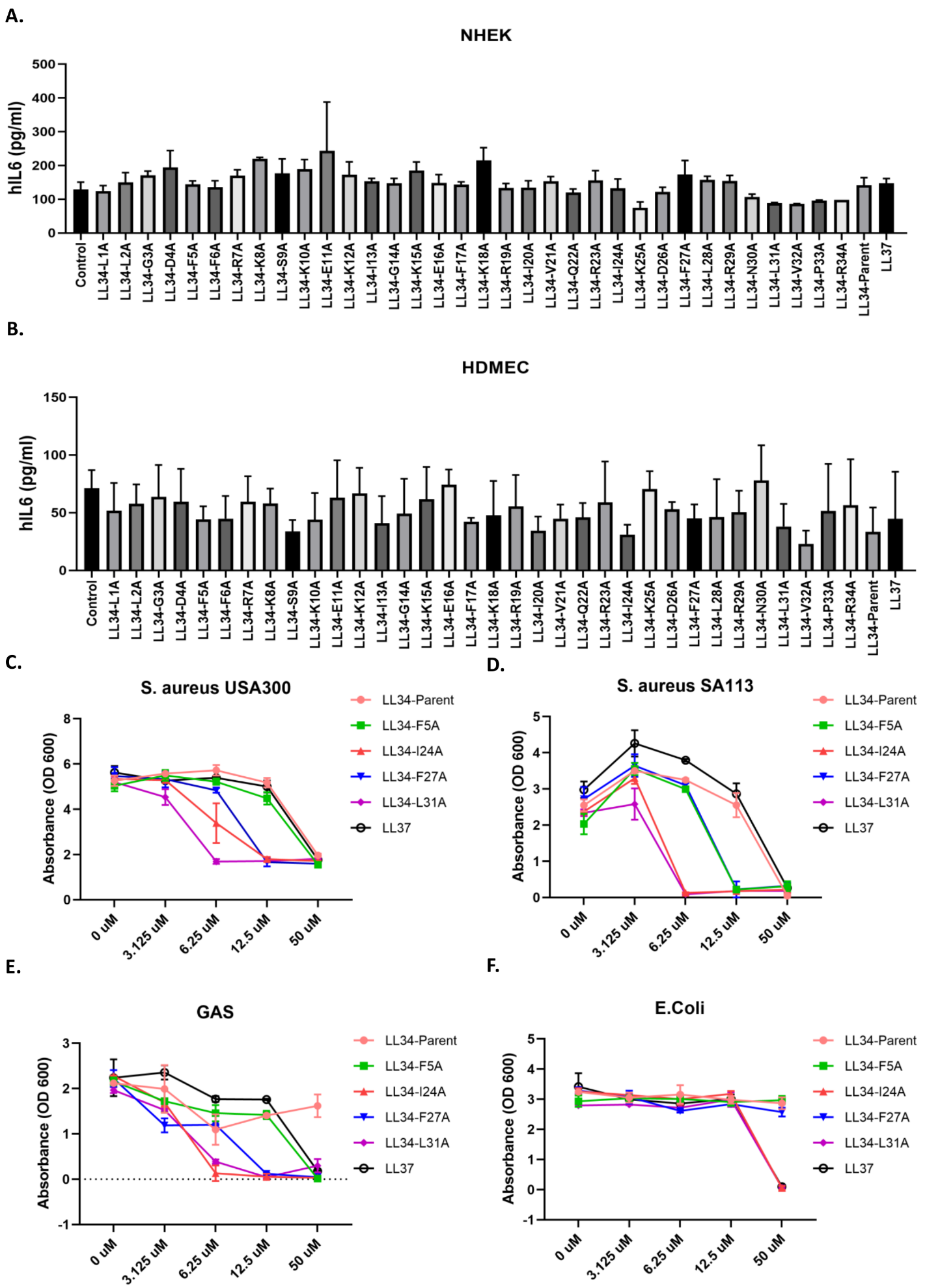


#### Supplemental Figure S1: LL34 alanine substitution differentially affects bacterial growth inhibition

#### (A) NHEKs and HDMECs (B) were treated with LL34- parent peptide and all the 34 alanine substituted peptides (Pep L1A-Pep R34A) and LL37 at 2 µM for 16 h. IL-6 protein secretion was assessed with ELISA (n=3) (C-F) LL34-Parent, LL34 F5A, LL34 I24A, LL34 F27A, and LL34 L31A peptides were incubated with *S. aureus* USA 300, *S. aureus* SA113, *Group A Streptococcus* (GAS) and *E. coli* at increasing concentrations (0-50 µM) overnight, and bacterial culture growth was assayed as absorbance at OD 600 (n=3).

####
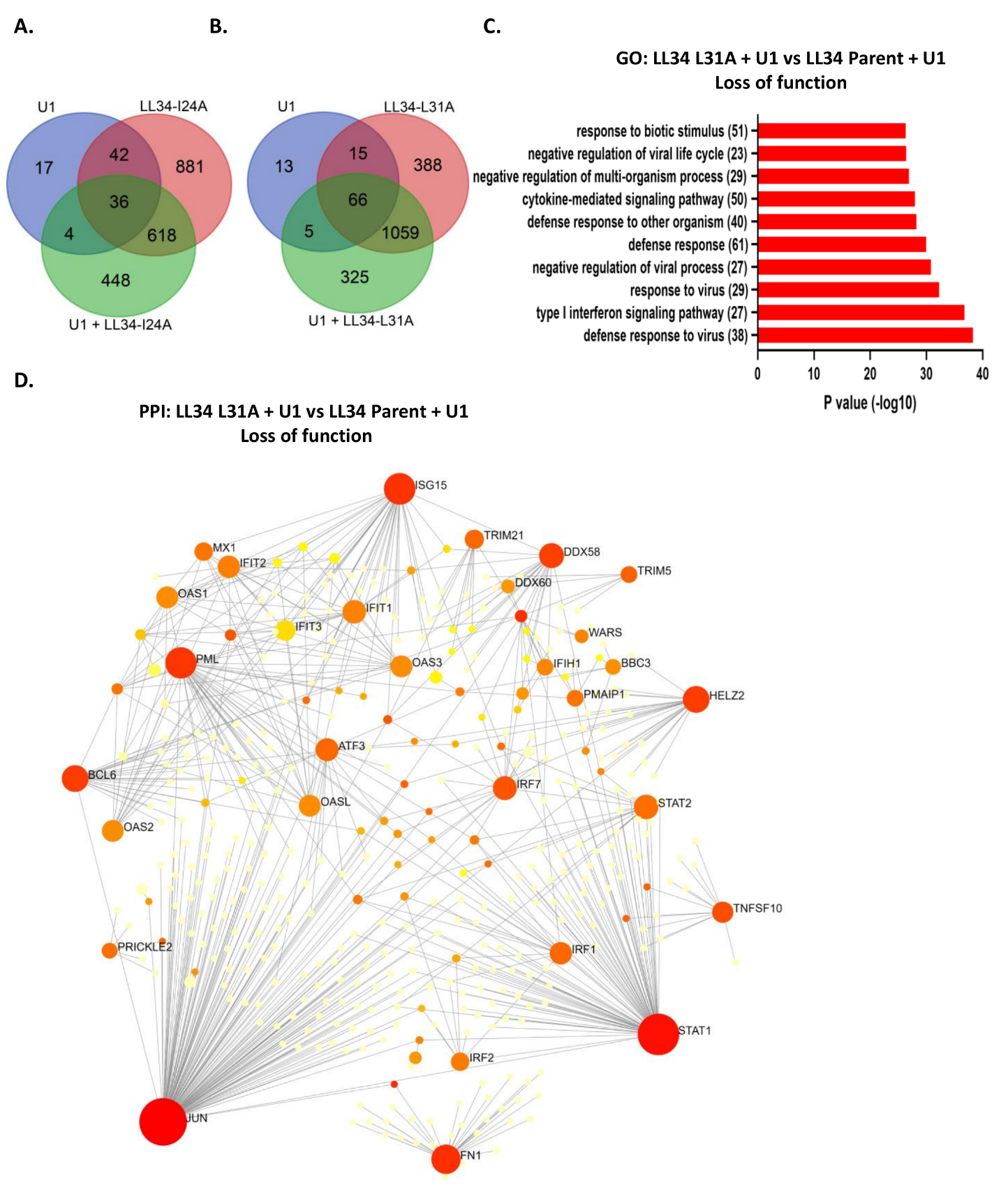


#### Supplemental Figure S2: Transcriptional profiling reveals immune pathways differentially affected by I24A and L31A substitution of LL34

#### (A,B) Venn Diagram for gene set from NHEKs treated with a combination of U1 dsRNA, LL34 L31A, LL34 I24A and combination of either U1 dsRNA and LL34 L31A or U1 dsRNA and LL34 I24A as shown (C) Gene ontology (GO) pathway analysis of pathways down-regulated/repressed in cells co-treated with U1 dsRNA and LL34 L31A vs U1 dsRNA and LL34-parent peptide and (D) Protein-Protein interaction networks were derived from cells co-treated with U1 dsRNA and LL34 31A mutant peptide and compared with cells treated with U1 dsRNA and LL34 Parent. Red Seeds indicate genes form over-represented networks and are indispensable.

####
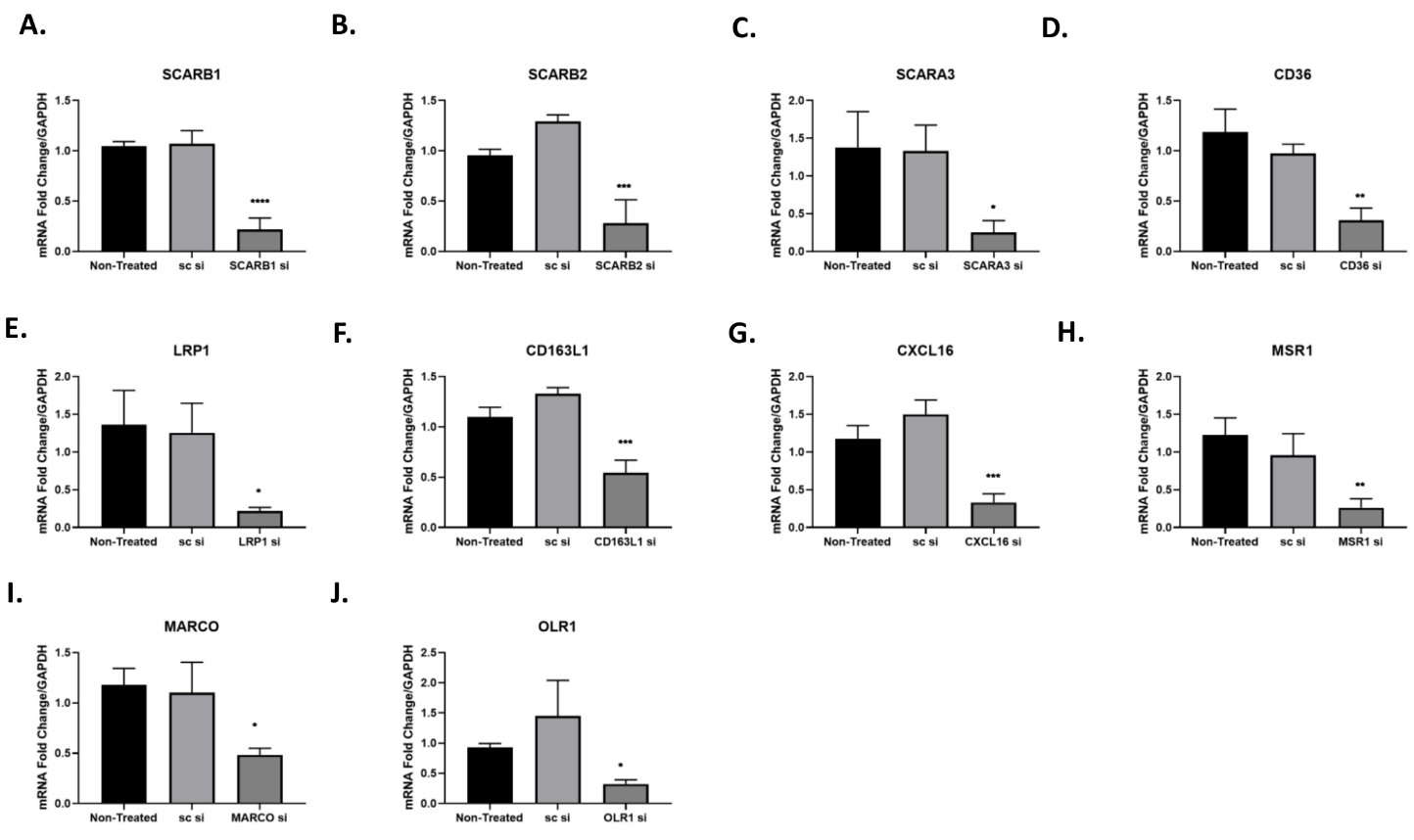


#### Supplemental Figure S3: Scavenger receptor knockdown verification (A-J)

#### NHEKs were transfected with siRNA against scavenger receptors SCARB1, SCARB1, SCARA3, CD36, LRP1, CD163L1, CXCL16, MSR1, MARCO and OLR1 for 24 h and gene knockdown was assessed with q-RT PCR (n=3, One-way ANOVA). (*, p< 0.05; **, p<0.01; ***, p<0.001; ****, p<0.0001)
